# Prepartum Lying Behavior of Holstein Dairy Cows Housed on Pasture through Parturition

**DOI:** 10.3390/ani7040032

**Published:** 2017-04-14

**Authors:** Christa A. Rice, Nicole L. Eberhart, Peter D. Krawczel

**Affiliations:** Department of Animal Science, University of Tennessee, Knoxville, 2506 River Dr. 258 Brehm Animal Science Knoxville, Knoxville, TN 37996, USA; ckurman@utk.edu (C.A.R.); neberhar@vols.utk.edu (N.L.E.)

**Keywords:** transition cow, lying behavior, pasture

## Abstract

**Simple Summary:**

Dairy cows experience meaningful biological changes during gestation that impact cow comfort and alter behavior, particularly during late gestation and leading up to calving. The housing environment can also have a positive or negative effect on cow comfort. Pasture access allows cows the freedom of movement and an ability to express natural grazing and resting behaviors. After observing cows housed on pasture during the late gestation and calving periods, this study found that lying behaviors only differed on the day of calving and the day prior to calving. Additionally, the proportion of time spent lying per hour decreased in the hour prior to calving compared to 6 h prior to parturition. The altered lying behaviors and activity observed in the hours before calving may indicate a decrease in cow comfort experienced by the cow during parturition. However, discomfort is typical of parturition. These data suggest that cows were able to express natural behaviors associated with calving and pasture when provided an adequate environment for cows during the prepartum period.

**Abstract:**

Utilizing pasture-based systems may increase cow comfort during late gestation and calving as it lacks the constraints of confinement housing. The objective of this study was to quantify lying behavior and activity of Holstein dairy cows housed on pasture during the 6 d before calving. Sixteen Holstein dairy cows were moved to pasture 3 weeks before their projected calving date. Data loggers were attached 14 d prior to projected calving date. Behavior was evaluated 6 d before calving for all cows (*n* = 16) and 6 h prior to calving for a subset of cows (*n* = 6) with known calving times. Data loggers recorded at 1-min intervals to determine lying time (h/d and %/h), lying bouts (n/d and n/h), lying bout duration (min/bout), and steps (n/d and n/h). A repeated measures analysis of variance with contrasts was performed to determine if lying behaviors and activity differed between baseline and day or hour of interest. Lying time was greater 6 d prior to calving compared to the day of and before calving. Cows had longer lying bouts 6 d prior to calving compared to day of calving. Cows spent less time lying in the hour before calving compared to 6 h prior to parturition. The lack of change in behavior and activity during the 7 d prior to calving may indicate that pasture provided an adequate environment for cows during the prepartum period but did not impact cow welfare in the hours leading up to calving.

## 1. Introduction

A dairy cow experiences inherent stressors during the late stages of gestation, however, providing an appropriate housing environment may help alleviate the behavioral impact of these stressors. Housing options for late gestation dairy cows in the United States vary widely by producer [1]. This variation in housing indicates that there exists no consensus on the best approach for managing dairy cows during this stage of lactation.

In 2014, dairy cow management data was collected via interviews and questionnaires from dairy producers [1]. Pasture access for dry cows was common with 72.3% of operations allowing dry cows access to pasture (11.3% of those operations used pastures as a primary housing system for dry cows) and 34.0% of dry cows having some access to pasture [1]. For operations relying on indoor housing for dry cows, 18.2% of operations used tie stalls or stanchions with no outside access, 9.5% utilized freestalls without outside access, and 20.3% used freestalls with outside access [1].

The current understanding of dairy cow behavior surrounding calving is based on data from confinement housing [2,3,4]. Cows housed in freestalls spent approximately 10–11 h per d lying which was consistent over 10 d before and after calving [2]. Cows housed in individual calving pens 4 d prior to calving exhibited a decrease of 104 min/d in lying time and an increase of 0.8 bouts/d in lying bouts when compared to the 24 h before calving [3]. A competitive feeding situation tended to decrease lying time during the week before calving with competitively fed cows lying for 494 min/d vs. non-competitively fed cows which spent 641 min/d lying [4]. The number of standing bouts increased to 17.3 bouts/d during the 24-h period before and after calving relative to the mean number of bouts during the 10 d before and after calving (11.7–13.1 bouts/d) [2].

The greatest changes in behavior around calving occurred in the 24 h before and after parturition. Lying time decreased by approximately 2 h and standing bouts increased during this period [2] compared to pre- and post-calving periods. Cows housed in individual calving pens increased their lying bouts and overall activity during the 6 h before calving [3]. Calving difficulty also influenced behavior during this time; cows who calved unassisted increased their lying bout frequency over the 6 h before parturition, but cows who needed assistance only increased their lying bout frequency 2 h before parturition [5]. 

Many dairy producers have begun to recognize the benefits of pasture at a variety of stages of the lactation cycle. These benefits include decreased lameness and reduced restriction to natural behaviors, such as lying down, by freestall hardware [6,7]. Lying or resting behavior is an essential component of cow comfort and is an indicator of animal welfare. Cows housed indoors spend approximately 12 h per day lying or resting [8] and cows housed on pasture spend approximately 9 h per day engaging in lying behaviors [9]. Overall, cows prefer soft bedding surfaces and stall dimensions that minimize contact with the hardware of freestalls [10,11,12]. Pasture offers a soft surface and is free of restrictive freestall hardware, making it easier for cows to lie down comfortably. This suggests pasture may alleviate some of the inherent discomfort during the late gestation period, as indicated by the laterality that occurs throughout this period [13]. More importantly for the context of this study, the pasture-based system may reflect the preference of prepartum dairy cows. When given a choice between a freestall barn or pasture mid-lactation, cows spent less than 30% of their time on pasture between the morning and evening milkings, but spent 90% of their time on pasture at night [14]. Since the cows in this study were mid-lactation animals, it is likely that their feeding and milking routines affected where they chose to spend time, especially during the day [14]. Despite the confounding effects of feeding and milking routines, these data support the previous hypothesis that pasture may be more comfortable for cows than confinement systems, making it an ideal option for housing cows in the prepartum period because cows often experience stress and discomfort during this time [15].

Despite the common use of pasture in the dry period, to our knowledge, all research to date on lying behavior in transition cows has been performed in a confinement or freestall setting while preference for pasture was only assessed in mid-lactation animals. The objective of our study was to quantify the lying behavior and activity of Holstein dairy cows housed on pasture during the 6 d before calving and for the 6 h before calving for a subset of cows. It was hypothesized that lying behavior and activity would be unaffected before calving due to the improved ability of free movement provided by the pasture.

## 2. Materials and Methods

### 2.1. Animals, Housing, and Measurement

All cows were housed at the University of Tennessee’s East Tennessee Research and Education Center (Walland, TN, USA). Sixteen Holstein dairy cows going into their second or greater lactation with a mean 305 ME of 12,380 kg ± 702.1 kg and mean 245 ± 5.5 DIM during previous lactations were moved from freestall housing to pasture approximately 3 weeks before their expected calving date. Behavior and activity data were collected from the 16 cows for 6 d before calving. A subset of 6 cows from the original 16 cows with known calving times were used to determine behavior 6 h before calving. The study took place from March to May with cattle on pasture being maintained as a dynamic group. The cows were housed in the same pasture, but not simultaneously. As groups of cows approached day 42 (42 days into the dry period), they were moved from the freestalls to the pasture. Pasture population ranged from a minimum of 2 cows to a maximum of 18. Cows were moved to pasture in groups of 10 (including cows not enrolled in the study) on a weekly basis as they were close to calving and removed from the pasture group immediately after calving. Cows in the pasture were checked approximately eight times per day by farm staff.

The pasture was 7.9 acres of orchard grass and fescue. Cows were moved a distance of 167 m by way of a fenced drover’s lane from the barn to the pasture. Supplemental feed offered within the pasture consisted of round baled wheat, rye grass, or Sudan grass, and refusal TMR from the lactating herd. Round bales were fed as needed on a concrete feed pad which was 6 × 15 m with six concrete feed bunks and a hay rack measuring 2 × 3 m. Water was available ad libitum from an automated waterer surrounded by gravel located near the center of the pasture. Cows were able to seek natural shade in two areas (0.07 acres and 0.27 acres, respectively). Cows were checked multiple times throughout the day and during feeding. The University of Tennessee IACUC (protocol #1982-0111) approved all animal procedures. 

### 2.2. Data Collection

IceTag data loggers (IceRobotics Ltd., Edinburgh, Scotland, UK) were attached to the cow’s hind legs approximately 14 d before the expected calving date to collect data on lying time (min/d), number of lying bouts (bouts/d), duration of lying bouts (minutes/bout), and steps/d. The use of IceTag loggers for this type of research was previously validated [16]. A lying bout was defined as ≥2 min of uninterrupted lying [17]. For the subset of six cows, data loggers were used in the same manner and proportion of the hour spent lying (%/h), number of lying bouts (bouts/h), and steps/h were evaluated for 6 h prior to calving. Because some lying bouts lasted longer than an hour, they were excluded from the hourly analysis. For the daily analysis, data was summarized with day beginning at midnight.

### 2.3. Data Analysis

The individual cow was the experimental unit for all analyses. A repeated measures analysis of variance with contrasts using SAS (v9.3, SAS, Inc., Cary, NC, USA) was performed to determine if lying behaviors and activity differed between baseline and day or hour of interest. For evaluation of the daily means, the behavioral responses on day-6 was used as a baseline and the mean response of each subsequent day was compared against it. For the evaluation of hourly means, the behavioral responses of lying time (min/h) and steps/h were compared to the response 6 h prior to calving. Significance was declared at *p* ≤ 0.05.

## 3. Results 

### 3.1. Lying Time

**Figure 1:**
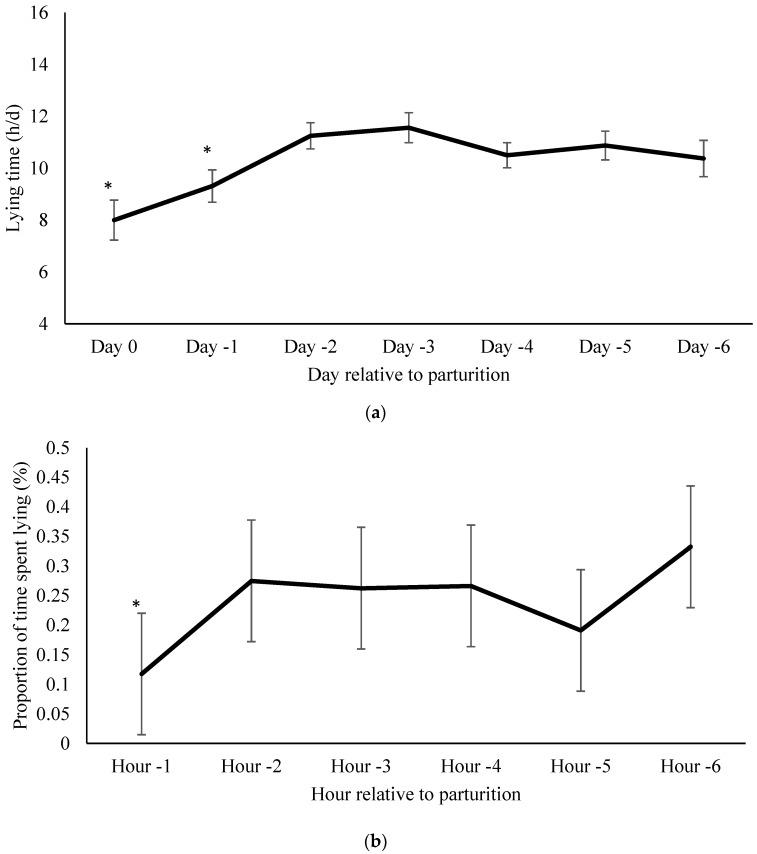
(**a**) Lying time per day relative to parturition. Changes in average lying time in minutes one week before calving in 16 cows. All days were compared to day-6 as a baseline (mean ± SE). * indicates *p <* 0.01. On the days of and before calving cows spent less time lying down compared to d-6. * indicates *p* < 0.05; (**b**) Proportion of time spent lying per hour relative to parturition. Mean hourly lying time 6 h before calving in six cows (mean ± SE). All hour intervals were compared to 6 h prior to calving as a baseline. In the hour before calving (hour −1), cows spent less time lying than 6 h prior to calving (*p* = 0.0360). * indicates *p* < 0.05.

Mean daily lying time of cows was (mean ± SE) 10.3 ± 0.3 h/d in the 6 days prior to calving. Lying time decreased to 9.3 ± 0.6 the day before calving and to 8.0 ± 0.8 the day of calving (*p* = 0.006 and *p* = 0.009, respectively; Figure 1a). Cows in the subset spent a mean proportion of 24.0% ± 4.1% of each hour lying during the 6 h before calving. Proportion of lying decreased in the hour prior to calving (11.8% ± 5.6%, *p* = 0.04; Figure 1b).

### 3.2. Number of Lying Bouts

**Figure 2:**
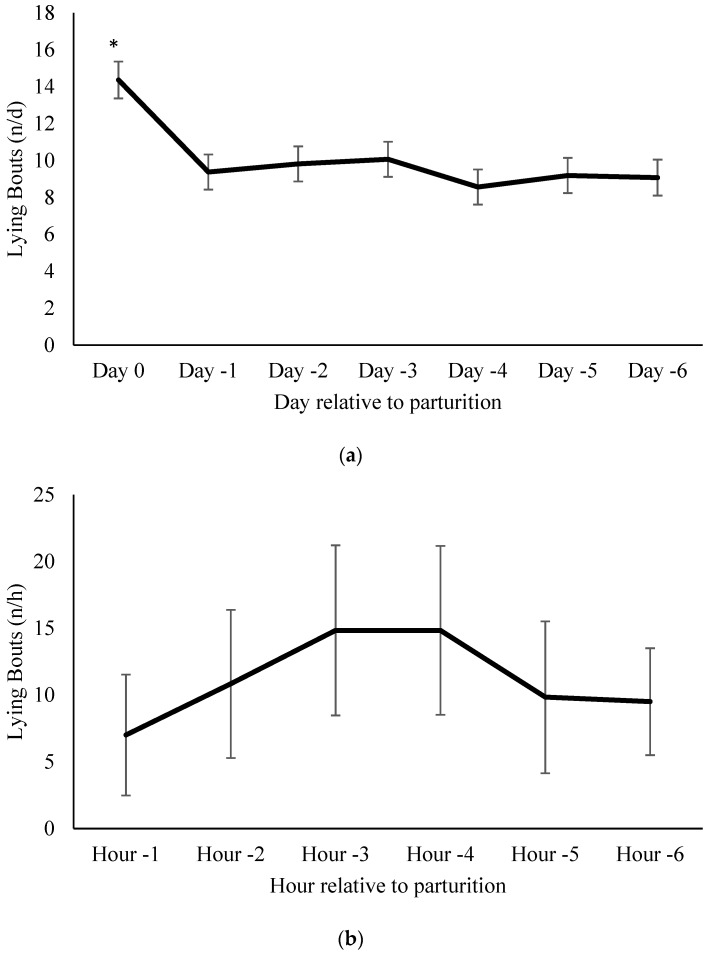
(**a**) Lying bouts per day relative to parturition. Mean number of lying bouts one week before calving in 16 cows (mean ± SE). All days were compared to day-6 as a baseline. On the day before calving cows had a greater number of lying bouts compared to day-6; * indicates *p* < 0.05; (**b**) Lying bouts per hour relative to parturition. Mean hourly lying bouts 6 h before calving in six cows (mean ± SE). There were no differences in lying bouts between the baseline (−6 h) and the hours leading up to parturition.

Cows in the current study had a mean daily number of lying bouts of 10.0 ± 0.4 n/d throughout the study. Lying bouts did not differ in the days leading up to parturition (*p* ≥ 0.07; Figure 2a). For the subset of cows, mean number of lying bouts during the 6 h before calving was 11.8 ± 2.2 bouts/h. The number of lying bouts did not differ in the hours leading up to parturition (*p* ≥ 0.2987; Figure 2b).

### 3.3. Lying Bout Duration

**Figure 3:**
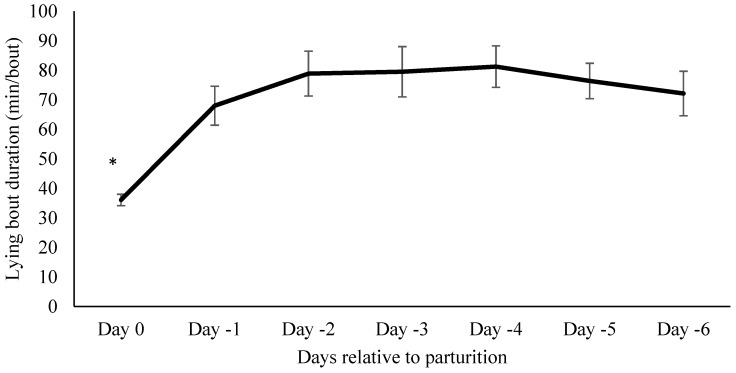
Lying bout duration in the days leading up to parturition. Mean lying bout duration in minutes one week before calving in 25 cows (mean ± SE). All days were compared to day-6 as a baseline. Lying bouts were shorter on the day of calving compared to day-6. * indicates *p* < 0.05.

Mean lying bout duration in the days leading up to calving was 96.9 ± 14.9 min/d. Lying bout duration was shorter the day of calving compared to day-6 (*p* < 0.0001; Figure 3).

### 3.4. Steps

**Figure 4:**
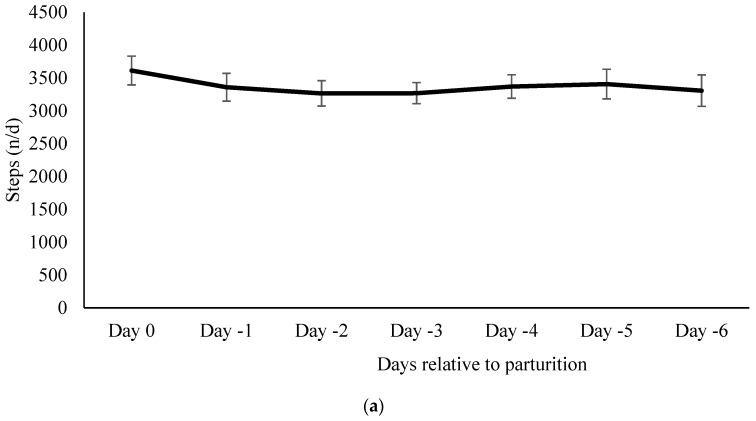

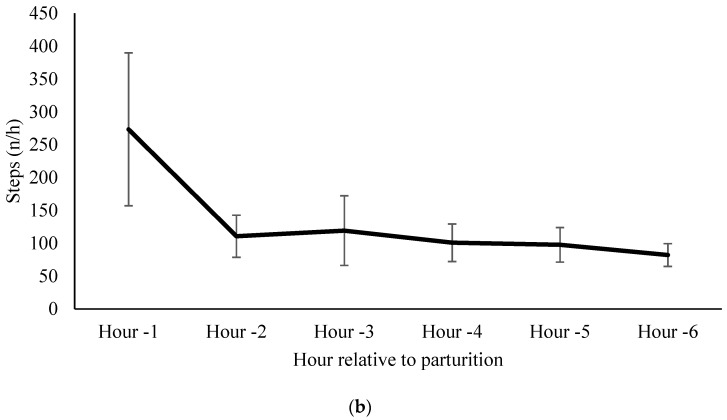
(**a**) Steps per day relative to parturition. Mean number of steps one week before calving in 16 cows (mean ± SE). All days were compared to day-6 as a baseline. There were no differences in the number of steps in the 7 d before calving compared to day-6; (**b**) Steps per hour relative to parturition. Mean number of steps 6 h before calving in six cows (mean ± SE). All hour intervals were compared to the baseline (6 h before calving). There were no differences in activity in the hours leading up to parturition.

Cows engaged in a mean of 3369.1 ± 76.0 steps/d during the days before calving. The number of steps did not differ between the days before calving (*p* ≥ 0.48; Figure 4a). During the 6 h before calving for the subset of cows there was an average of 142.8 ± 7.7 steps/h. Steps/h did not differ during the 6 h period before calving (*p* ≥ 0.18; Figure 4b).

## 4. Discussion

### 4.1. Lying Time

The observed lying time response was consistent with lying time reported for primiparous and multiparous cows fed in non-competitive conditions, where lying time ranged from approximately 600 to 650 min/d [4]. In contrast, values from the current study were considerably lower than the mean responses of 738 min/d and 985 min/d reported for cows housed in confinement over the last 10 or 4 d before calving [2,3]. When comparing freestall and pasture, cows on pasture had the lowest daily lying times (approximately 660 min/d) while cows confined to freestalls had the highest daily lying times (around 780 min/d) [14]. These data indicate that the lying times observed in the current study are within the normal range for cows on pasture. 

Jensen observed that cows lay down less during the 6 h period before calving when compared to 2–4 days before calving [3]. Although cows on the previous study had greater mean daily lying time than cows in the current study, these data may suggest that pasture had no negative effect on prepartum cow comfort in conjunction with reduced lying times. 

### 4.2. Number of Lying Bouts

Mid to late lactation cows on pasture had more lying bouts/d (15.3 bouts/d) than prepartum cows in the current study (10.0 ± 0.4 bouts/day) [6]. However, the previous study focused on cows with lameness, indicating that the greater number of lying bouts per day could have been due to decreased comfort associated with the lameness. Similarly, during the day before calving, confinement housed dairy cows increased their standing bouts which was attributed to discomfort and restlessness during parturition [2]. The lack of increase in lying bouts in the current study during the day before calving indicate that cows housed on pasture were less restless and uncomfortable during this time than cows housed in confinement during the same period. This suggests future work on the merits of prepartum housing may need to focus on lying bouts rather than lying time to assess cow comfort.

### 4.3. Lying Bout Duration

Lying bout duration was generally close to 60 min/bout across several studies of behavior of cows approaching parturition within confinement housing (63 min/bout, [2]; 57 min/bout, [3]; 50 min/bout, [5]). While the bout duration in the current study was considerably longer, it was within the range of 43 dairy farms in British Columbia, Canada (50 to 118 min/bout) where lying behavior was assessed in mid-lactation cows [8]. This may indicate that even though cows were approaching calving they did not experience enough discomfort to affect lying bout duration. It has been hypothesized that a reduction in lying time due to discomfort from events, such as an uncomfortable lying surface, was driven by a reduction in lying bouts rather than changes in bout duration [11]. 

### 4.4. Steps

Cows did not alter activity in the week prior to calving, which was in contrast to data obtained from animals housed in a confinement setting where cows increased activity during the day before calving [3]. Similarly, no change in the number of steps for cows housed on pasture in the 6 h prior to calving may indicate that pasture housed cows experience less restlessness before calving. Pain may cause an increase in activity since acute pain such as that associated with the induction of mastitis caused an increased number of steps [18]. However, cows on pasture spend more time grazing than cows in confinement spend feeding, suggesting that activity differences between confinement and pasture may not be similar. 

## 5. Conclusions

Cows housed on pasture may experience reduced restlessness and discomfort associated with calving. When on pasture, cows did not show signs of discomfort, such as increased activity or increased number of lying bouts. Cows on pasture may also be more comfortable standing, as indicated by lower lying times when compared to literature values for cows housed in confinement. Further research to compare groups of cattle housed on pasture and confinement is needed in order to determine if pasture provides improved comfort for prepartum dairy cattle.
